# Navigation Experience and Mental Representations of the Environment: Do Pilots Build Better Cognitive Maps?

**DOI:** 10.1371/journal.pone.0090058

**Published:** 2014-03-06

**Authors:** Jennifer E. Sutton, Melanie Buset, Mikayla Keller

**Affiliations:** Department of Psychology, Brescia University College, London, Ontario, Canada; University of Sussex, United Kingdom

## Abstract

A number of careers involve tasks that place demands on spatial cognition, but it is still unclear how and whether skills acquired in such applied experiences transfer to other spatial tasks. The current study investigated the association between pilot training and the ability to form a mental survey representation, or *cognitive map*, of a novel, ground-based, virtual environment. Undergraduate students who were engaged in general aviation pilot training and controls matched to the pilots on gender and video game usage freely explored a virtual town. Subsequently, participants performed a direction estimation task that tested the accuracy of their cognitive map representation of the town. In addition, participants completed the Object Perspective Test and rated their spatial abilities. Pilots were significantly more accurate than controls at estimating directions but did not differ from controls on the Object Perspective Test. Locations in the town were visited at a similar rate by the two groups, indicating that controls' relatively lower accuracy was not due to failure to fully explore the town. Pilots' superior performance is likely due to better online cognitive processing during exploration, suggesting the spatial updating they engage in during flight transfers to a non-aviation context.

## Introduction

The potential for spatial cognitive abilities to improve through targeted spatial training in the laboratory, and for those trained abilities to transfer to other spatial tasks, is well-established (see [1] for a recent meta-analysis), but less is known about how experience in the applied settings of spatially-demanding careers affects spatial cognition. Perhaps the best known example of changes due to career experience is London, UK taxi drivers, who learn the complex layout of London and show increased hippocampal volume with driving experience [2]–[4], coupled with a cognitive pattern suggesting enhancement in some areas, such as route learning in a new town [5], and decrements in others, such as incorporating a new area into their existing representation [3]. This mixed pattern of results is echoed in work with individuals training in other careers that involve the acquisition of smaller-scale spatial visualization skills. For instance, dental students, who learn to visualize cross-sections of teeth, do not perform better on novel object cross-section tests or other general spatial ability tests at the end of training [6], while engineering students' spatial visualization scores do seem to improve over the course of undergraduate study [7]. Taken together, it is currently unclear how the spatial skills gained in the course of training in applied situations transfer to other tasks and activities that also recruit spatial abilities.

Some data suggest that aviation experience may be associated with improvement in spatial abilities. Dror, Kosslyn, and Wagg [8] compared the small-scale spatial abilities of established military pilots and non-pilot undergraduates and found that pilots showed better mental rotation ability than non-pilots, although no difference was found on other tests such as judging categorical spatial relations and image scanning. Several aspects of the study prevent strong conclusions about the effect of flight experience on spatial cognition, however. First, the pre-training selection process for military pilots includes tests of small-scale spatial ability [9], so it is unclear whether time spent flying, and not superior pre-existing spatial abilities, would be the critical factor in military pilots' superior performance on mental rotation. Second, small-scale spatial abilities are related to but distinct from the larger-scale spatial demands of navigation such as spatial updating and the incremental buildup of a mental representation of an environment [10]. The tests of Dror et al. isolated small spatial components of navigation, and tests of other, larger-scale spatial processing abilities are required to gain a more complete picture of how flight might influence spatial processing.

Despite the limitations of the Dror et al. [8] study, pilots still provide a unique opportunity to investigate the effect of applied experience on spatial cognition. In particular, pilots taking flight lessons in *general aviation* (i.e., non-military, non-commercial-airline flight) are not pre-selected on the basis of test scores and are therefore likely provide a more representative sample of the general population than military pilots for investigations of the effects of experience. Like taxi drivers, these pilots spend their working time navigating and attending to the technical aspects of operating a complex machine, but unlike taxi drivers, they fly to and from a variety of widely-spaced locations, and they experience a unique aerial viewpoint. Aerial views of the earth play an important role in aircraft navigation, especially early in a pilot's career when flight is restricted to clear weather with visibility greater than 5 miles (i.e., Visual Flight Rules, or VFR). VFR navigation is accomplished primarily by looking out the window at landmarks on the ground with the aid of a map [11], [12]. Aretz [13] described pilots as having “navigation awareness” during flight when they mentally represent both where landmarks are relative to the plane using an egocentric reference frame and where landmarks are relative to each other in a “world-centered”, or allocentric, reference frame, also known as pilotage or wayfinding [14], [15].

The allocentric mental representation of an environment that navigation awareness requires is also known as a survey representation or *cognitive map* [16], [17]. This type of mental representation can be contrasted with a route-based representation of an environment that consists of memory for the sequence of turns and landmarks encountered when traversing a specific path [18]. While it is still debated whether a cognitive map develops only after experience with a number of routes [18] or develops in parallel with route knowledge [19], researchers do agree on two aspects of the representation. First, a cognitive map of an area is a more sophisticated and flexible representation than one based on routes, as it can be used to integrate information from separate routes [18], [19] and allows the traveller to plan and take novel short-cuts between landmarks [20]. Second, there are clear differences across individuals in the tendency and ability to form a cognitive map representation based on similar exposure to an area [19], [21].

In the current study, we were interested in whether pilots, who engage in cognitive mapping to maintain navigation awareness during flight, would form more accurate cognitive maps of an unfamiliar ground-based virtual environment relative to non-pilots. We gave university-student general aviation pilots and university-student non-pilot controls a period of self-guided, active exploration in a novel virtual town. The accuracy of participants' mental representations of the town was then assessed using a judgment of relative direction (JRD) pointing task, e.g., [22], which required individuals to imagine a heading between two landmarks in the town and then point in the direction of a third landmark. This task is difficult to perform using a route-based representation [21], and greater accuracy indicates a more accurate cognitive map. Participants also completed the paper-and-pencil Object Perspective Test (OPT) [23], a perspective-taking task similar to the JRD but without the memory requirement, allowing us to isolate pointing ability from spatial memory. Finally, participants completed the Santa Barbara Sense of Direction Scale (SBSOD) [24], a measure of self-perceived spatial abilities.

We predicted that pilots would show greater accuracy on the JRD and OPT than matched controls. We were also interested in whether pilots' cumulative flight hours would be associated with better accuracy on these tests. Finally, we were interested in whether self-perceptions of spatial abilities reported on the SBSOD would match relative performance on the tasks.

## Method

### Participants

Thirty-six students at the University of Western Ontario participated in the study. Pilots (*n* = 18; 15 males, 3 females; mean age = 21.22, *SD* = 2.05, range = 19–26) were students who had at least 1 hour of flight experience in an airplane (*M* = 123.09 hours, *SD* = 83.10, range = 1–259) and reported being currently engaged in flight training. Twelve pilots held Private Pilot licences and three of those also held a Commercial licence. All pilots were enrolled in the Commercial Aviation Management (CAM) undergraduate program at the university in which they take general and aviation-related business management courses as part of a business degree and earn, over the course of years 2–4, a Commercial Pilot licence via flight instruction at a local flight school. The Control group participants (*n* = 18, mean age = 22.22, *SD* = 2.71, range = 19–28) were matched to the pilots on the basis of participant sex and self-reported frequency of video game usage (see [25] for a review of video game experience and spatial cognition), except one pair for whom the pilot's game frequency rating was 4 and the matched control participant's rating was 3 (see below for details on the rating scale). In total, 49 control participants were tested; tests were scored and data were analyzed and are reported only for the 18 participants who matched a pilot participant on the relevant criteria.

#### Ethics Statement

This research was approved by the University of Western Ontario Department of Psychology Research Ethics Board, a sub-board of The University of Western Ontario's Research Ethics Board for Non-Medical Research Involving Human Subjects.

### Materials, Equipment, & Procedure

Participants provided informed consent in written form and then completed an initial questionnaire where they provided information about hours of flight experience, aviation licenses and ratings held, video game usage and, for those who reported playing video games, how frequently they played (rated from 0 = *less than 1 time per week* to 5 = *5 or more times per week*) along with the names of games they played. Next, as preparation for the nonimmersive virtual reality town task, participants practiced moving around in a virtual room unrelated to the main task using the joystick controller (Logitech Extreme 3D Pro, Logitech, Newark, CA) and the Windows laptop with a 15.6″ widescreen display (Samsung R525, Samsung Electronics, Suwon, South Korea), an AMD Phenom II Quad-Core N970 2.2 GHz Processor and an AMD Radeon HD 6600M Graphics card (Advanced Micro Devices, Sunnyvale, CA).

**Figure 1:**
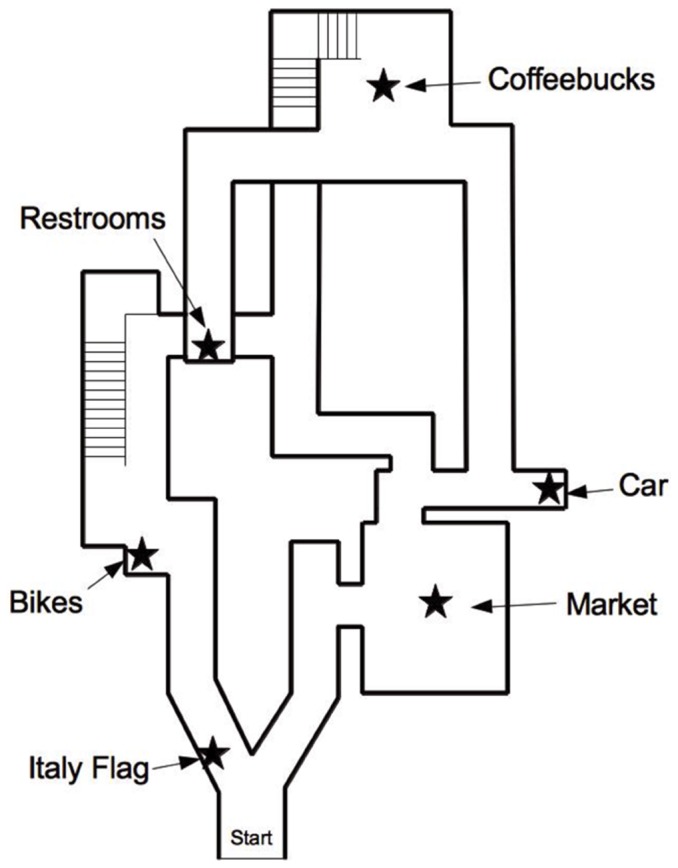
Overhead view of the virtual town. Streets were lined by tall buildings which prevented visual access from one route to another except at intersections. One exception is the restrooms which were located on an upper-level breezeway.

After an unlimited amount of practice using the joystick, participants were given 5 minutes to freely explore the virtual reality town. Figure 1 shows an overhead view of the town layout. Both the virtual town and the practice rooms were created using the Half-Life 2 game engine and the Source Software Development Kit (Valve Software, Bellevue, WA). The town was modified from an older virtual game town (“Italy” map for the game Counter-Strike, Valve Corporation) by introducing new objects, simplifying the routes through the town, and restricting access so that participants could not travel inside buildings. Tall buildings lined the streets in the town. Overall, the town was 4153 (North to South)×1513 (West to East) virtual units, where each unit corresponded to a perceived size of approximately 1.9 cm; therefore, the town occupied a perceived area of 2.18 km. The participants' apparent eye-level was 64 virtual units, or 1.21 m, above the ground with a 75° horizontal field of view. Participants were asked during debriefing if they recognized the town and none did; in addition, Counter-Strike was not listed as a game played by any participant on the questionnaire. The town included 6 distinct locations; a list of these locations was provided for participants on a sheet of paper for the duration of the exploration period: flag, market, bikes, old car, coffeebucks coffee shop, and restrooms. Participants were instructed to find all the locations during the exploration period in order to draw a sketch map later, a manipulation shown to encourage cognitive map formation [10], [26].

After the exploration period, participants completed a 12-item judgment of relative direction (JRD) task based on their memory of the layout of the town. Each item was presented as text on the laptop screen outside the virtual environment without any images from the town present. Each item prompted participants to point (using the joystick) to estimate a direction after being prompted with a town location and a heading direction (for instance, *Imagine you are at the market facing the restrooms. Point to coffeebucks*.). Each item was represented twice as the initial position, the facing location, and the target, except the market which served as the target once and the flag which served as the target three times in order to balance the number of correct headings that were in regions to the front left, front right, back left, and back right of the participant. Following the JRD task, participants drew a sketch map of the town. Anticipation of these sketches served as motivation for forming a survey representation of the town [26], but they were not analyzed since it is unclear how they relate to mental representations of space [27]. Next, participants rated their navigation abilities on the SBSOD [24] and finally completed the paper-and-pencil OPT [23], a paper-and-pencil test of perspective taking that requires participants to provide a heading estimate similar to the JRD task but without the memory component, as the same configuration of seven objects is present for every item. Each item requires participants to draw an imagined heading on a circle, similar to the pointing response in the JRD (e.g., *Imagine you are standing at the car and facing the traffic light. Point to the stop sign*). Participants are given 5 minutes to complete as many items as possible.

## Results

### Gaming

Participants in both groups reported playing video games an average of twice a week. Of the participants that played video games and listed the games they played, the majority in both groups listed at least one game that falls in the “Action” category [25] and therefore requires substantial spatial processing (pilots: 14/14 games listed, controls: 12/14, although one control participant reported “strategic”, which may or may not have a action/spatial component and is excluded from this calculation). Perhaps not surprisingly, one noticeable difference in game playing between the groups was the prevalence of flight simulator activity by pilots who played games (50%) versus controls who played games (7%).

### Direction estimates

**Figure 2:**
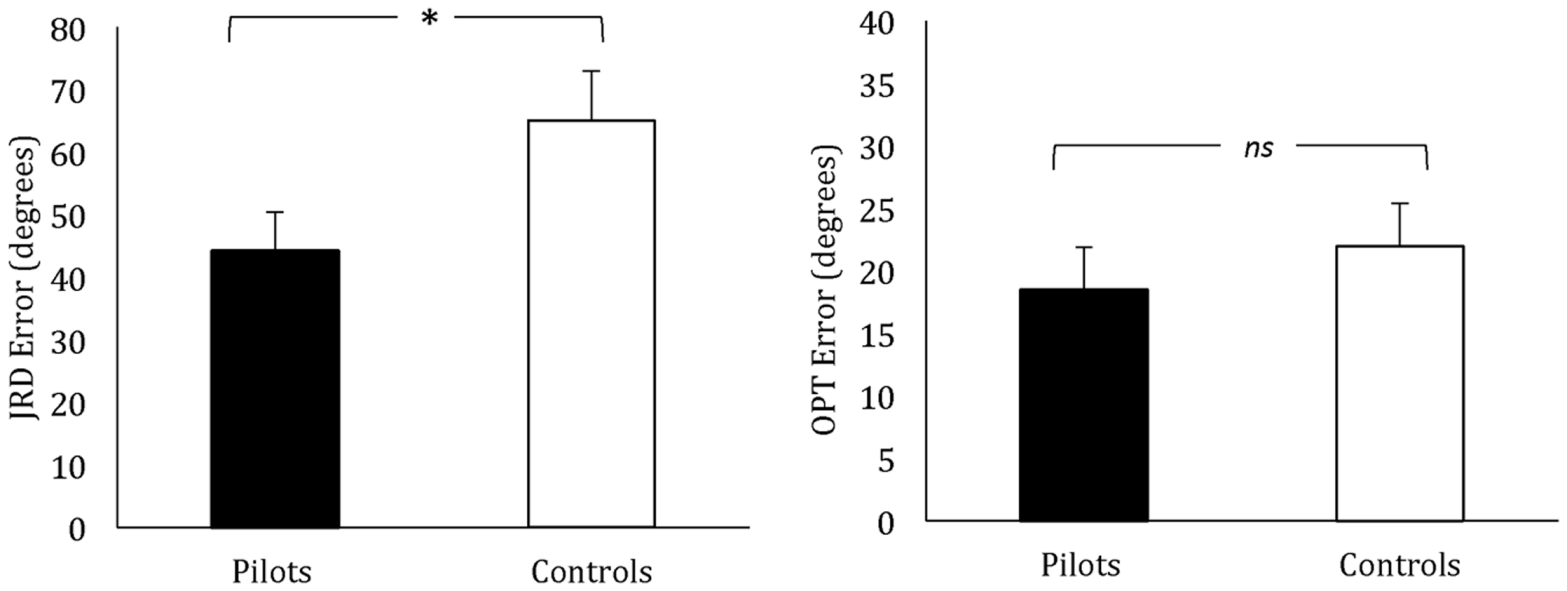
Performance of pilots and non-pilot controls on the spatial measures. Mean error in absolute degrees is shown for pilots and non-pilot controls on the Judgment of Relative Direction task (JRD) and the Object Perspective Test (OPT). Lower error indicates greater accuracy. Error bars show standard error of the mean. *p = .04.

Accuracy on the JRD task based on free exploration of the virtual town was scored as absolute error in degrees of the participant's response heading from the actual heading on each item. As can be seen in Figure 2, pilots' error (*M* = 44.23, *SD* = 26.29) was significantly lower than control participants' error (*M* = 65.04, *SD* = 33.89), *t*(34) = 2.06, *p* = .047, Cohen's *d* = .71. There were no outliers (individual's mean score or average score for a particular location >3 standard deviations from the mean) in either the control or pilot group.

Accuracy on the OPT was also scored as absolute error of the participant's response in degrees away from the correct heading. Here, however, there was no significant difference between pilots (*M* = 18.47, *SD* = 14.36) and controls (*M* = 21.96, *SD* = 14.58), *t*(34) = .72, *p* = .47, *d* = .25 (right panel of Figure 2).

**Figure 3:**
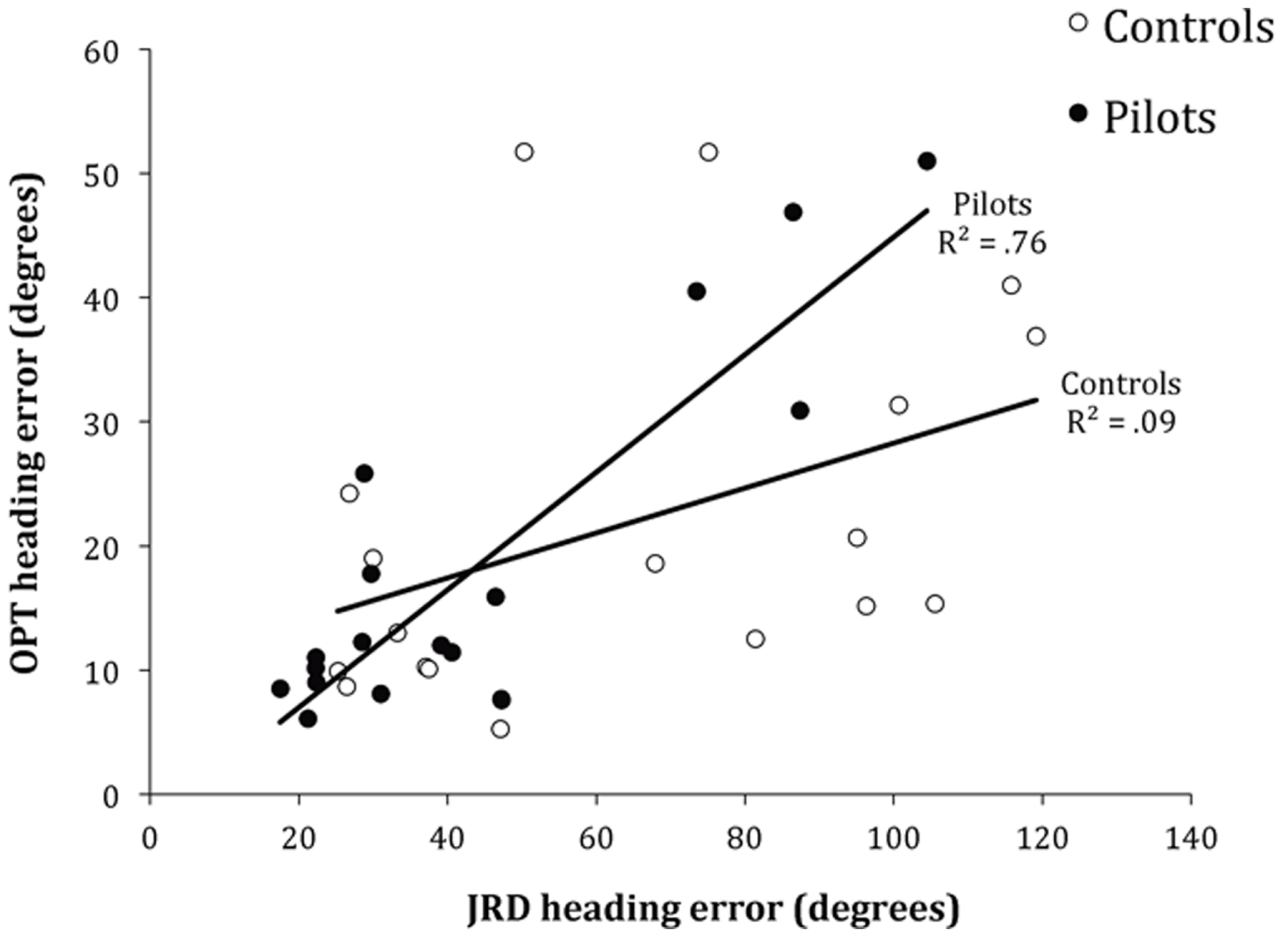
Association between the two spatial measures. Association of Judgment of Relative Direction (JRD) scores and Object Perspective Test (OPT) scores for pilots and non-pilot control participants.

In addition, associations between scores on the OPT and JRD tasks were analyzed via a series of Pearson correlations. Over all participants, JRD and OPT scores were significantly correlated, *r*(34) = .61, *p*<.001. As can be seen in Figure 3, however, this correlation appears to be driven mostly by the pilots, as the correlation of their scores on the two tasks was strong and significant, *r*(16) = .87, *p*<.001, but scores for the control group were not significantly correlated, *r*(16) = .42, *p* = .08.

For the pilots only, Pearson correlations were also conducted to investigate the association between hours of flight experience and performance on the spatial measures. The correlation between flight hours and performance on the OPT was significant using a one-tailed test, *r*(16) = −.42, *p* = .04, (two-tailed test, *p* = .08), indicating a tendency for pilots with more hours to show less error in their heading estimations. There was no significant correlation between flight hours and performance on the JRD task in our sample, *r*(16) = −.13, *p* = .61.

### Location visits during town exploration

To explore whether pilots' increased JRD performance was related to how often participants in both groups visited locations in the town, number of visits to each location in the town, with a visit defined as being within 343 virtual units from the center of a location (6.5 meters), were analyzed in a Group (pilots, controls)×Location (flag, market, bikes, old car, coffeebucks, restrooms) mixed ANOVA. There was no Group×Location interaction, Greenhouse-Geisser corrected *F*(3.68, 125.05) = 0.43, *p* = .77, η^2^ = .003, indicating that the pilots and controls did not differ in frequency of visits across the different locations. The two groups also did not differ in average visits to all locations in the 5-minute period (pilots: *M* = 6.39, *SD* = .24; controls: *M* = 5.93, *SD* = .24), as there was no main effect of Group, *F*(1, 34) = 1.99, *p* = .17, η^2^ = .004. There was a significant main effect of Location indicating that participants in both groups visited some areas more often than others, Greenhouse-Geisser corrected *F*(3.68, 125.05) = 88.84, *p*<.001, η^2^ = .66. Bonferroni-corrected posthoc paired *t* tests showed that visits to coffeebucks (*M* = 10.47, *SD* = 2.87) and the market (*M* = 8.78, *SD* = 2.10) did not differ significantly, *p* = .08, but both were visited more often than the bikes (*M* = 6.31, *SD* = 2.56), the car (*M* = 6.06, *SD* = 1.77), the restrooms (*M* = 2.36, *SD* = 1.22) and the flag (*M* = 3.00, *SD* = 1.49), *p*s for all comparisons <.01. The restrooms and the flag were visited less often than all other locations, *p*s<.01, and did not differ significantly, *p* = 1.0. Visits to the bikes and the car did not differ significantly, *p* = 1.0, the car was visited more frequently than the restrooms and the flag, and the bikes were visited more often than the restrooms and the flag, all *p*s<.01. Importantly, this pattern was the same for both groups of participants, as evidenced by the lack of a Group×Location interaction.

### Self-ratings of spatial ability

On the SBSOD questionnaire, participants rated their spatial ability from 1–7; some items were reverse scored so that 7 indicates highest ability and 1 indicates lowest. Pilots rated their spatial ability (*M* = 5.55, *SD* = .56) significantly higher than controls (*M* = 4.32, *SD* = 1.49), *t*(21.65) = 3.29, *p* = .003, Cohen's *d* = 1.41 (Levene's test indicated heterogeneity of variance, *F* = 10.90, *p* = .002, therefore degrees of freedom were adjusted from 34 to 21.65 for the *t* test). For the pilots, self-rated spatial ability on the SBSOD was significantly negatively correlated with error on the OPT test, *r*(16) = −.56, *p* = .02, but not the JRD test, *r*(16) = −.39, *p* = .12. Control participants' self-ratings were not related to error on the OPT, *r*(16) = −.36, *p* = .14 but just approached significance for the JRD task, *r*(16) = −.45, *p* = .06.

## Discussion

As predicted, undergraduate student pilots were more accurate at estimating directions between landmarks in a virtual town (the JRD task) after self-guided exploration than matched non-pilot controls. The matching procedure for the control group effectively eliminated gaming and sex as drivers of this effect. The difference between the groups on the JRD task was not due to control participants having difficulty finding all the locations while exploring the town, since both groups showed a similar frequency and pattern of visits to town locations. Contrary to predictions, there was no difference between groups on the OPT, indicating that the pilots were not better at perspective taking and/or pointing per se. Performance on the JRD and OPT tasks was correlated for pilots but not controls, and pilots' hours of flight experience was related to performance on the OPT but not the JRD task. Finally, pilots gave their own spatial abilities higher ratings than controls gave theirs, and pilots' ratings corresponded with their performance on the OPT.

The superior performance of pilots on the JRD task and the similar exploration patterns with controls suggests that pilots formed more accurate cognitive map representations than controls, who were either more likely to rely on a route-based representation or on cognitive maps that were less accurate. Creating an accurate cognitive map based on self-guided exploration, as in the current task, places heavy demands on visuospatial working memory and executive function: Participants must build, maintain, and update a survey representation while simultaneously planning and controlling movement [10], [28]. It is interesting that pilots' performance on the OPT and JRD were related although control participants' scores on the tests were not. The format of the items on the tests is similar, and it makes sense that perspective taking ability would factor heavily in performance on both; controls who were accurate on the OPT seem not to have leveraged their perspective-taking ability to perform consistently well on the JRD items, however. This suggests that the increased accuracy of pilots on the virtual town JRD was based on better encoding, maintenance, and updating of a mental map during town exploration, which may be an instance of transfer of skills acquired in aviation to this novel environment.

Even though the pilots, as a group, performed more accurately on the JRD task than controls, we did not find clear evidence in our sample of a direct association between flight hours and accuracy on the JRD task. There was, however, an association between hours and performance on the OPT. The association of hours of experience and perspective taking, plus the association of perspective taking and performance on the JRD task suggests that flight experience may affect the formation and retrieval of a cognitive map representation by way of improved perspective taking. Allen, Kirasic, Dobson, Long, and Beck (1996) [29] found some support for perspective taking ability as a mediator of general spatial ability and accuracy on a heading estimation task similar to the JRD. Further tests are warranted, but the current data suggest pilots' cognitive mapping accuracy may be due to perspective taking skills that develop with flight experience.

In summary, these results suggest that pilots, through their navigation experience, are better at forming cognitive maps of a novel (non-flight) environment than non-pilots. On the other hand, it is possible that individuals who choose to learn to fly in general aviation have better spatial abilities than non-pilots to begin with. It could also be that a more definitive positive association of flight hours and spatial abilities in non-flight environments emerges in pilots after many more hours of flight. These two possibilities are not mutually exclusive, so a longitudinal design incorporating a control group is the ideal way to further investigate how spatial abilities improve as flight experience increases. In addition, studies designed to address the structure of the mental representations pilots form and the kinds of strategies they consciously employ (for instance, with reference to cardinal directions), will help specify just how flying experience changes spatial cognitive processes. The current data suggest that even in the initial stages of learning to fly in a general aviation context, changes are occurring in pilots' mental representation of space and that such a longitudinal study is worthwhile.

An accurate mental representation of the spatial environment is central to flying [30], even with the increased information and automation provided by advanced cockpit avionics systems which have, somewhat paradoxically, not led to increased safety in general aviation [31]. For instance, the use of GPS when flying under VFR appears to decrease navigation awareness and introduces the potential for an unsafe situation should the equipment fail [30]. Therefore, pilots in general aviation need to commit substantial cognitive resources to processing spatial information and provide an excellent population to further investigate the effects of applied experience on spatial cognition.
